# Case report: Plastic bronchitis associated with *Bordetella parapertussis*

**DOI:** 10.1097/MD.0000000000034239

**Published:** 2023-07-07

**Authors:** Zhongjie Li, Yao Xu, Weirong Shen

**Affiliations:** a Department of Pediatrics, Jiashan No. 1 People’s Hospital, Jiashan, China.

**Keywords:** *Bordetella parapertussis*, case report, plastic bronchitis, treatment

## Abstract

*Bordetella parapertussis* caused by a severe infection is rare in clinical practice. Here, we report a case of plastic bronchitis (PB).

**Patient concerns:**

A 4-year-old girl with a 2-day history of fever, paroxysmal cough, and subconjunctival hemorrhage.

**Diagnoses:**

The diagnoses were (1) *B parapertussis*, (2) pulmonary atelectasis, and (3) PB.

**Interventions:**

The patient received azithromycin and underwent bronchoscopy.

**Outcomes:**

Symptoms disappeared after treatment. The patient had an outpatient follow-up of 2 months without respiratory symptoms.

**Lessons:**

PB can lead to respiratory failure if not intervened in the early stages.

## 1. Introduction

*Bordetella parapertussis* can cause the same pertussis-like symptoms, which are responsible for only 2% to 20% of cases and usually cause less severe pulmonary disease.^[1,2]^ Case studies of severe illnesses associated with *B parapertussis* are rare, with only 3 cases,^[3,4]^ 2 of which were aware of the underlying conditions. Therefore, severe clinical features of *B parapertussis* may be associated with impaired or decreased immune function in the host. Herein, we describe a case of *B parapertussis* associated with plastic bronchitis (PB). The etiology of PB includes surgery for congenital heart disease, infection, inflammation, and allergies. Infection is one of its main causes. PB is an unusual and severe pulmonary disease characterized by the presence of mucous casts in the tracheobronchial tree, which can fill the trachea and lead to progressive respiratory failure. These therapies use bronchoscopic checkups to extract bronchial casts.

## 2. Patient presentation

**Figure 1.**
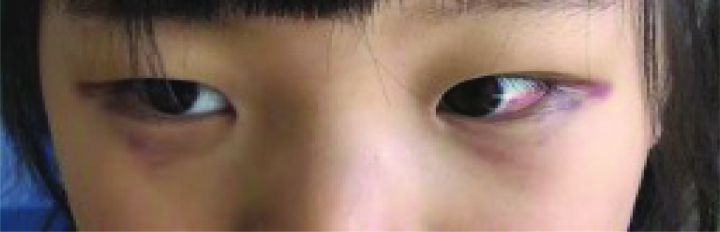
The bilateral subconjunctival hemorrhages and eyelids swelling with lower eyelids hemorrhages.

**Figure 2.**
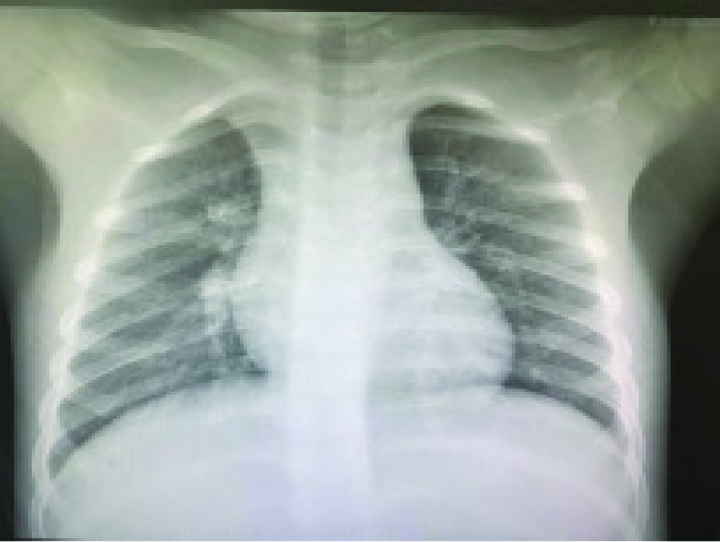
X-ray showing lung marking changes.

A 4-year-old girl was admitted to the Department of Pediatrics with a 2-day history of fever and mild dry cough in December 2021. Her parents heard mild paroxysmal cough occasion in night. Several times, she came close to post-tussive vomiting. She had no shortness of breath, dyspnea, postnasal drop, and reflux. On examination, she was lethargic, weight 17.0 kg, the temperature was recorded as 38.6 °C, heart rate 120 beats/min, respiratory rate of 26 breaths/min, oxygen saturations was 96% on room air, and capillary refill was <2 seconds. The bilateral subconjunctival hemorrhages and eyelids swelling with lower eyelids hemorrhages (Fig. 1). The tonsils were prominent but without active inflammation or exudate, neck lymphadenopathy was noted. Air entry was good throughout and chest was moist crackles on auscultation. Peripheral blood test, including full blood count and C-reactive protein, were normal (hemoglobin 133 g/L, white blood count 9.1 × 10^9^/L, platelets 145 × 10^9^/L, lymphocytes, and neutrophils) and C-reactive protein 8.36 mg/L. A chest radiograph shows bronchitis (Fig. 2).

## 3. Past medical history

The girl was born preterm at 36 weeks of age via a normal vaginal delivery and weighed 2 kg at birth. She had no developmental delay or other complications, was not breastfed, and was fed a formula from birth. Childhood immunizations have been performed on schedule and are currently available. The patient had no history of asthma, allergy, lung disease, tobacco use, or substance use and had no other abnormal exposures or history and recent travel. The parents and their 9-year-old son remained well, with no fever, cough, or other respiratory symptoms.

## 4. Treatment

**Figure 3.**
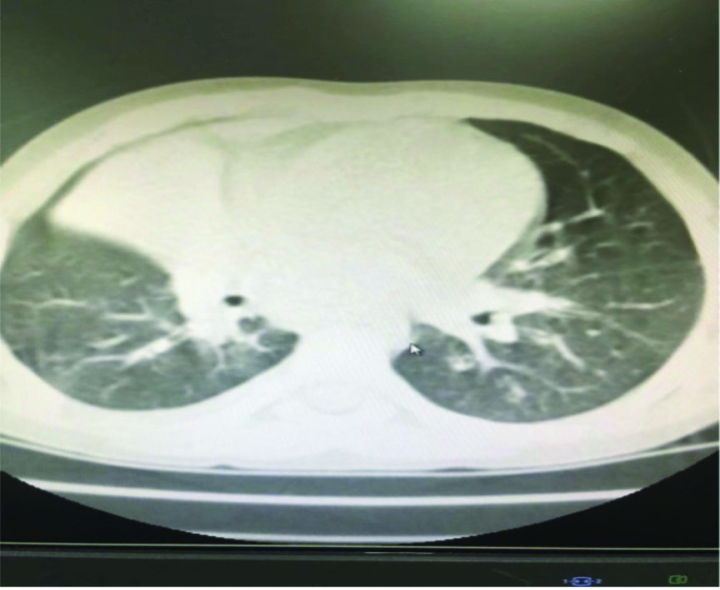
Computed tomography (CT) scan showing right middle lobar consolidation.

**Figure 4.**
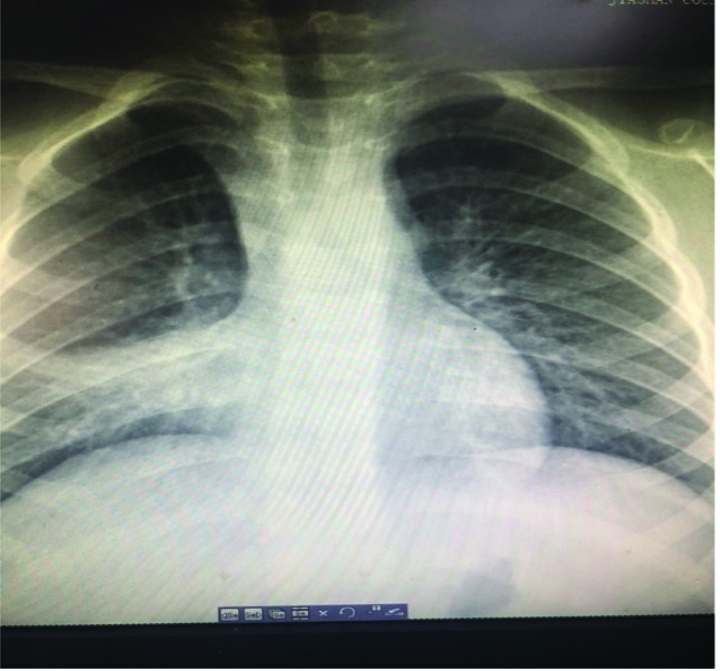
X-ray showing laminar atelectasis in the right middle lobe.

**Figure 5.**
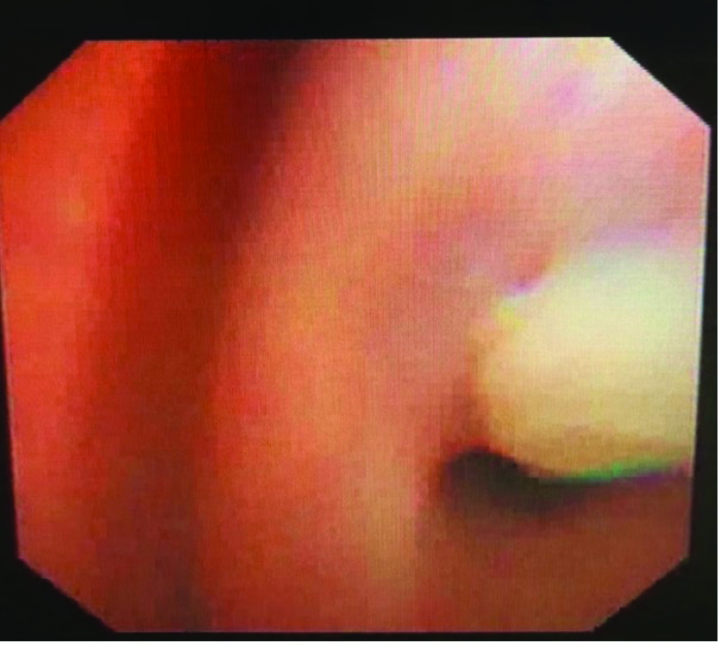
Flexible bronchoscopy, which revealed for a whitish rubbery material occluding the right middle of robe bronchus.

**Figure 6.**
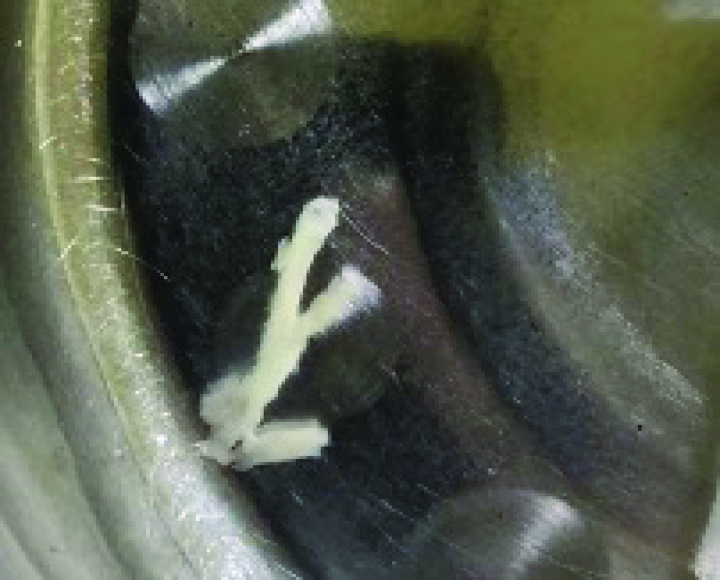
Cast removed from the right middle of the bronchi.

**Figure 7.**
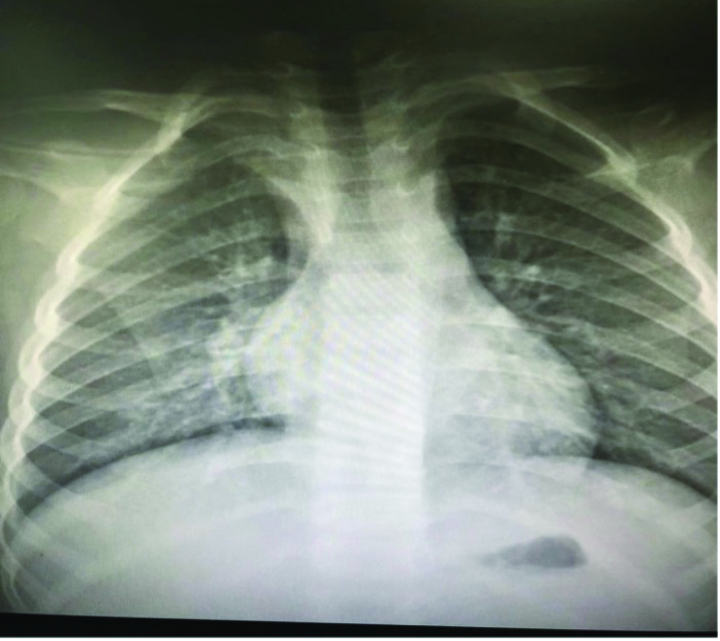
X-ray showing increased lung marking.

During hospitalization, the patient developed a persistent fever and paroxysmal cough. Computed tomography revealed a right middle lobar consolidation (Fig. 3). The patient was started on fluid therapy and intravenous ceftriaxone 1.3 g/d on day 3. Because of subconjunctival hemorrhage, eyelid swelling, paroxysmal cough, and post-tussive vomiting, pertussis was diagnosed after consultation, azithromycin (10 mg/kg) was added to ceftriaxone.^[5]^ On day 7, the fever, cough, and eye symptoms improved. Her D-dimer level was middling at 3250 μg/L. The liver and kidney function test results were normal, ferritin 792.64 ng/mL (reference: 4.63–204), and her Epstein-Barr (EB) antibodies were as follows: anti-VCA IgM antibody, >160 (normal: <20); Epstein-Barr virus (EBV) anti-VCA IgG antibody, <10 (normal: <20); and EBV anti-EBNA IgG antibody, negative. Influenza A and B antigens were negative, *Mycoplasma pneumoniae* antigen was negative, and hepatitis B surface antigen and hepatitis C antibody test results were negative. The human immunodeficiency virus antigen/antibody test result was negative (cut-off index < 1.0). *Treponema pallidum* hemagglutination was negative. The blood culture was a sterile ultrasound showing a normal liver, spleen, kidney, and gallbladder wall edema. Radiography revealed laminar atelectasis in the right middle lobe (Fig. 4). The patient still had a fever, and atelectasis continued without improvement. Bronchoscopy was performed to check the condition of the pulmonary bronchi. Flexible bronchoscopy revealed that off-white colloidal material occluded the right middle lobe bronchus (Fig. 5). The plastic casts were then removed (Fig. 6). The operation was performed under general anesthesia, with no adverse or unexpected events, and the patient’s or unexpected events, and the patient’s tolerance was good. Targeted next-generation sequencing: *B parapertussis* and human gammaherpesvirus 4 (EB) were detected in the bronchoalveolar lavage fluid. The ceftriaxone treatment was discontinued. As the patient had no definite signs of hematologic neoplasms or immunodeficiency, we chose to closely monitor and not administer any specific therapy for the EBV infection. On day 10 (the day 4 after flexible bronchoscopy) the patient’s general condition improved and respiratory symptoms disappeared. Radiographs revealed increased lung markings (Fig. 7). Antibiotic therapy was discontinued, and the patient was discharged. The patient had an outpatient follow-up of 2 months without respiratory symptoms.

## 5. Discussion

PB is a rare and insufficiently diagnosed disease characterized by the presence of mucous casts in the bronchial lumen, which can be life threatening.^[6]^ In the pediatric population, PB is usually associated with cardiothoracic surgery, infections, inflammatory processes, acute chest syndrome, or iatrogenic processes.^[7]^ Infection is a common cause of PB. The most commonly reported pathogens are influenza viruses (A and B), *M pneumoniae*, adenovirus serotype 7, *Mycobacterium tuberculosis*, and fungi.^[8–11]^ Herein, we report a case of *B parapertussis* and human gammaherpesvirus 4 (EB). However, the mechanism underlying cast formation remains unclear. PB, which is caused by an infection, has an analogous pathogenesis. Currently, plastics are thought to be caused by multiple inflammatory cell infiltrations and inflammatory mediators as well as the consequences of hyperemia, edema, necrosis, and occlusion of the lumen of the tracheal mucosa.^[11]^ There is no clear inference regarding the pathogen that dominates the PB formation. Our results suggest that coinfection has a cooperative effect on PB formation. Early bronchoscopy intervention is helpful in shortening the length of hospital stay. Pathogen-targeted next-generation sequencing of the bronchoalveolar lavage fluid is useful for early diagnosis and treatment. Clinicians must be familiar with the typical clinical symptoms and the progression of the disease.

## 6. Conclusion

In conclusion, PB needs a treatment focused for removing or expectorating the cast, and implementing bronchoscopy techniques benefits patients.^[7]^

*B parapertussis* is endemic worldwide^[2]^and can cause outbreaks.^[5]^ Typical symptoms include a paroxysmal cough, post-tussive vomiting, and subconjunctival hemorrhage. *B parapertussis* can cause severe invasive infections,^[3,12]^ mostly in immunocompromised hosts.^[2]^ Patients with acute EB infection may be considered immunocompromised owing to their susceptibility to infection.^[13–15]^ Furthermore, her disease worsened, and shifted to PB. This study proposed that *B parapertussis* infection is a typical whooping cough. Studies have shown that *B parapertussis* can cause cough disease comparable to whooping cough and that pertussis vaccination cannot prevent it.^[16]^

## Author contributions

**Conceptualization:** Zhongjie Li, Yao Xu.

**Investigation:** Weirong Shen.

**Methodology:** Weirong Shen.

**Supervision:** Yao Xu.

**Writing – original draft:** Zhongjie Li.

**Writing – review & editing:** Zhongjie Li.
